# Non-Volatile Programmable Ultra-Small Photonic Arbitrary Power Splitters

**DOI:** 10.3390/nano12040669

**Published:** 2022-02-17

**Authors:** Huan Yuan, Jiagui Wu, Jinping Zhang, Xun Pu, Zhenfu Zhang, Yang Yu, Junbo Yang

**Affiliations:** 1 Center of Material Science, National University of Defense Technology, Changsha 410073, China; huanyuan1806@email.swu.edu.cn (H.Y.); zhangjinping@email.swu.edu.cn (J.Z.); zhenfuzhang@nudt.edu.cn (Z.Z.); yuyang08a@nudt.edu.cn (Y.Y.); 2 College of Electronic and Information Engineering, Southwest University, Chongqing 400715, China; 3 School of Physical Science and Technology, Southwest University, Chongqing 400715, China; 4 College of Computer & Information Science, Southwest University, Chongqing 400715, China; puxun@swu.edu.cn

**Keywords:** arbitrary power splitter, inverse design, phase change material, digital nanophotonics

## Abstract

A series of reconfigurable compact photonic arbitrary power splitters are proposed based on the hybrid structure of silicon and Ge_2_Sb_2_Se_4_Te_1_ (GSST), which is a new kind of non-volatile optical phase change material (O-PCM) with low absorption. Our pixelated meta-hybrid has an extremely small photonic integrated circuit (PIC) footprint with a size comparable to that of the most advanced electronic integrated circuits (EICs). The power-split ratio can be reconfigured in a completely digital manner through the amorphous and crystalline switching of the GSST material, which only coated less than one-fifth of the pattern allocation area. The target power–split ratio between the output channels can be arbitrarily reconfigured digitally with high precision and in the valuable C-band (1530–1560 nm) based on the analysis of three-dimensional finite-difference time-domain. The 1 × 2, 1 × 3, and 1 × 4 splitting configurations were all investigated with a variety of power–split ratios for each case, and the corresponding true value tables of GSST distribution are given. These non-volatile hybrid photonic splitters offer the advantages of an extremely small footprint and non-volatile digital programmability, which are favorable to the truly optoelectronic fusion chip.

## 1. Introduction

Perfect optoelectronic fusion chip solutions require photonic integrated circuits (PICs) and electronic integrated circuits (EICs) with two basic properties: (1) an extremely small PIC size and similar EIC size, enabling high integration; the difference in the order of magnitude between traditional PICs and EICs is a significant fusion barrier; (2) a PIC programmability that is also similar to EICs [1,2,3,4].

Power splitters are widely used in several applications as one of the very basic PIC devices [5,6,7]. They can be roughly classified into two types: those with a fixed proportional power–split ratio and those with an adjustable power–split ratio. In the past, multimode interferometers (MMIs) can be used as power splitters based on the principle of self-imaging in planar multimode waveguide [8] such as a 1 × 4 power splitter with two-stage cascaded MMI couplers connected by phase shifters [9] and 1 × 2 power splitters based on asymmetrical MMIs (by breaking the structural symmetry of multimode waveguide) [10,11]. Additionally, there are power splitters based on specific photonic crystal structures, such as a 1 × 2 power splitter with Y-shaped photonic crystals waveguides combined with point defects [12,13] and a 1 × 3 power splitter based on triangular lattice air hole silicon slab photonic crystals [14], that achieve good performance and excellent progress. However, this device’s design method adopts manual parameter adjustment technology and is limited to several parameters intuitively selected based on design experience. The size of the device remains large, ranging from tens of microns to hundreds of microns. To meet the demand for optoelectronic fusion chip solutions, inverse design methods have been rapidly developed in recent years. Different from the traditional forward design method, the inverse algorithm can discretize the pattern allocation area into only a few microns and realize various functions by constantly optimizing the refractive index distribution of parameters. This can effectively avoid using intuitive experience in device design and reduce the time cost. Examples include the direct binary search (DBS) algorithm [15,16,17], the genetic algorithm [18], and deep learning [19]. Recently, various ultra-small fixed-ratio power splitters based on inverse design have been reported. Xu et al. designed a device with a 3.6 × 3.6 µm^2^ footprint size with a QR code-like nanostructure and achieved 80% efficiency in their arbitrary power divider [20]. Tahersima et al. designed a multi-hole distributed nanostructure with an area of only 2.6 × 2.6 µm^2^ using a deep neural network, realizing multiple power–split ratios [21]. For such ultra-small devices with pixelated meta-structures, Huang et al. proposed the concept of digital nanophotonics [22]. Digital nanophotonic methods enable the design of complex refractive index distribution devices with a high degree of freedom and an ultra-small area [23,24,25,26]. This digitalized meta-structure is a photonic crystal-like (PhC-like) structure combining an MMI and a photonic crystal [16]. In the all-silicon MMI structure, the pores or materials are non-periodic and uneven. It has strong robustness to fabrication error, and the ultra-compact PhC-like structure can be an ideal choice for the inverse design of on-chip integrated photonic devices [22,27]. Compared with fixed-ratio power splitting, a power splitter with a variable power splitter ratio is also highly attractive and has a wide range of applications such as in feedback circuits and tap-port power monitoring. Tian et al. demonstrated a variable split-ratio power splitter on a silicon-on-insulator (SOI) architecture [28]. However, most of these silicon-based PIC devices exhibit a rather static (untunable) behavior.

Recently, optical phase-change materials (O-PCMs) have become highly attractive [29,30] by offering the feasibility of designing tunable photonic devices. Generally, O-PCMs can exist in either two states: crystalline or amorphous. The complex refractive index (real and imaginary) of an O-PCM varies significantly during the transition between amorphous and crystalline states, enabling the introduction of a large phase and amplitude modulation within the range of a compact device. This property has been exploited to explore PIC switching [31,32,33,34,35], reconfigurable element optics [36,37], photonic memory [38,39], and neural computing [40]. Moreover, experiments have proved that an O-PCM state is reversible from amorphous to crystalline and can be adjusted by absorbing the incident laser radiation or by electric heating [41]. The transition of the two states of an O-PCM should go through a multi-stage phase transition process so that performance is non-volatile. The formation of intermediate states can be promoted by adjusting the intensity of light pulses or electricity. These properties allow O-PCMs to maintain a constant light state even without power input as given in [29,42,43]; thus, it has optical non-volatility. However, most of the proposed power splitters do not have non-volatile adjustability and the size of the device is very large, usually a hundred times larger than that of EICs and, therefore, is not conducive for optoelectronic fusion. Thus, O-PCMs are a promising option.

In this study, we propose a non-volatile programmable photonic arbitrary power splitter using an all-digital nanophotonics design and O-PCM hybrid structure. The power–split ratio can be reconfigured in an all-digital way through the amorphous and crystalline switching of the non-volatile O-PCM material Ge_2_Sb_2_Se_4_Te_1_ (GSST). In the 1 × 2 splitter case, power ratios of 1:1, 1.5:1, 2:1, and 2.5:1 were achieved in the C-band (1530–1560 nm). Moreover, to show that the design method can be extended to a multi-channel splitter, a 1 × 3 (1 × 4) power splitter was studied with power–split ratios of 1:1:1, 2:1.5:1, 2:1:1, and 2:1:2 (1:1:1:1, 2:1:2:1, 3:1:2:1, and 2:2:2:1). All of these chips have an ultra-small footprint of 2.4 × 2.4 µm^2^ or 2.4 × 3.6 µm^2^, the same size level as EICs on chips, with orders of magnitude smaller than traditional silicon photonics devices. We believe that this ultra-compact and reconfigurable photonics platform provides an effective solution for designing a range of tunable photonic and truly optoelectronic fusion chips.

## 2. Principle and Simulation Results

**Figure 1:**
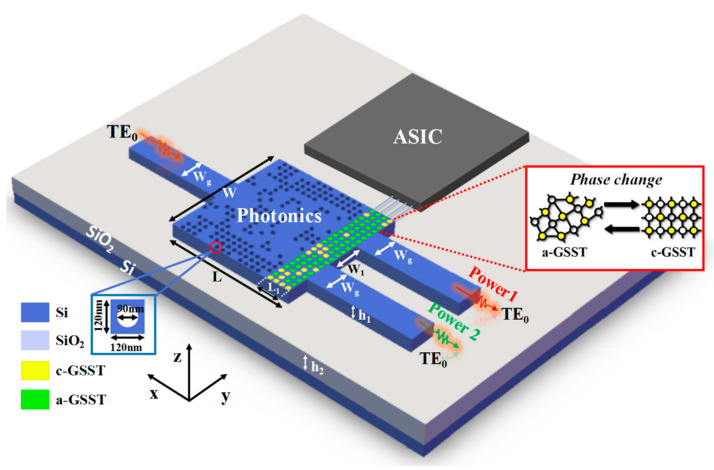
Schematic of the 1 × 2 photon programmable power splitter. Transverse electric (TE_0_) mode waves within a wavelength range of 1530–1560 nm were input from the left port. After these waves passed through the coupling region, the TE_0_ mode waves with Power1 and Power2 split ratios were output from the upper and lower channels on the right, respectively. The splitting ratio was regulated by the state of the phase-change material controlled by an electronic circuit program.

Figure 1 shows the functional concept of our programmable power splitter using silicon and O-PCM. The device consists of one input waveguide and one air hole, or a GSST-embedded silicon photonic waveguide coupling region and two output waveguides. By inputting a TE_0_ mode light into the device and controlling the state distribution (crystalline or amorphous) of the O-PCM, the device can dynamically achieve different power distribution ratios in the two output channels. All of the devices designed below supported only the TE_0_ mode. The silicon and O-PCM were combined using PhC-like subwavelength structures to realize the power splitter. Here, standard SOI waveguides were employed with a 220 nm thick silicon core layer and a 3 μm thick buried silicon dioxide layer [17]. The planar waveguide width was set at W = 2.4 µm (the width of both the input and output waveguides w_g_ was 500 nm, and the output waveguide spacing w_1_ was 600 nm), the length was L = 2.4 µm (the width of the GSST array region L_1_ was 480 nm), and the thickness was h_1_ = 220 nm (h_2_ = 3 µm). According to the fabrication process, the couple design area consisted of 20 × 20 pixels, each of which was a 120 × 120 nm^2^ square with a circular hole. The radius of the hole was 45 nm, and the depth of the hole was 220 nm. The GSST material could be embedded in these holes if necessary. In this design, different pixel states determine the distribution of the refractive index in the core region. We started with the all-silicon structure of an ordinary MMI. The fundamental transverse electric (TE_0_) mode light was emitted into the input waveguide, and a monitor could be used to measure the TE_0_ mode light power of the output waveguide. This structure can make full use of the free space so that the device has a good ability to regulate the light field in an ultra-compact size.

**Figure 2:**
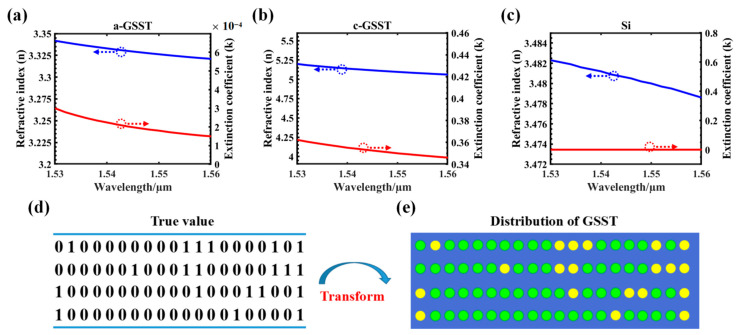
(**a**–**c**) Refractive indices, n, and the extinction coefficients, k, of a-GSST, c-GSST, and silicon from 1530 to 1560 nm; (**d**,**e**) GSST distribution corresponding to the true value.

As mentioned previously, the refractive index difference between the two states of O-PCM is quite large, and its nonvolatile phase transition enables stable phase or amplitude modulation. The most commonly used O-PCM is Ge_2_Sb_2_Se_5_ (GST), and it exhibits excessive light loss even in the dielectric state. There is a significant difference in the refractive indices of amorphous GST (a-GST; 1550 nm: 4.6 + 0.12i) and crystalline GST (c-GST; 1550 nm: 7.45 + 1.49i). However, the imaginary part of the refractive index in either state (a-GST or c-GST) is relatively high. If GST is embedded in the silicon structure to transmit light, it will cause excessive loss even in the amorphous state. To solve the problem of high GST loss, a new O-PCM, Ge_2_Sb_2_Se_4_Te_1_ (GSST), was developed to replace the traditional GST; a part of the Te in the traditional GST alloy was replaced by Se [42,43]. In the refractive index curves, shown in Figure 2a,b, the extinction coefficient of GSST in different crystal states was significantly lower than that of GST. At 1550 nm, the GSST complex refractive indices of the amorphous and crystallized states were n (a-GSST) + I × k (a-GSST) = 3.3258 + 1.8 × 10^−4^i and n (c-GSST) + i × k (c-GSST) = 5.0830 + 0.350i [43,44]. respectively.

A few GSST-based nanophotonic devices have been reported recently such as switches, modulators, and photonic memory [44,45,46]. Therefore, in this study, we introduced GSST as an O-PCM to construct a hybrid structure. The refractive index of amorphous GSST is quite close to that of silicon, so all the devices discussed herein used a-GSST as the initial pattern. O-PCM can be combined with silicon waveguides by placing it as a sheet on the waveguide surface or embedding it into the silicon hole structure. Here, GSST was embedded into silicon holes to effectively change the overall refractive index distribution [47,48,49]. Subsequently, the switching of O-PCM states can be realized digitally by optical or electric pulses [50,51]. As shown in Figure 1, the digital control can be realized by an on-chip ASIC (application-specific integrated circuit). Here, the ASIC function mainly controls the electric heating of the GSST unit in a program-controlled way. Using the logic circuit in the ASIC, it can connect the target light field distribution through the specific truth tables, which will be released in the follow-up results. Here, we introduce a hybrid structure, where the O-PCM was embedded in the silicon structure, thus achieving a significantly ultra-compact, nearly 10 times compression in a one-dimensional size and an approximately 100 times compression in a two-dimensional area compared to that of References [50,51]. Further, our O-PCM-coated area only occupied less than one-fifth of the pattern allocation area, with fewer control units and significantly easier ASIC handling. In particular, it should be noted that the ultra-compact photonics part was nearly the same size as the EICs’ part. Thus, this proposed scheme could be effective for the development of optoelectronic fusion chips.

**Figure 3:**
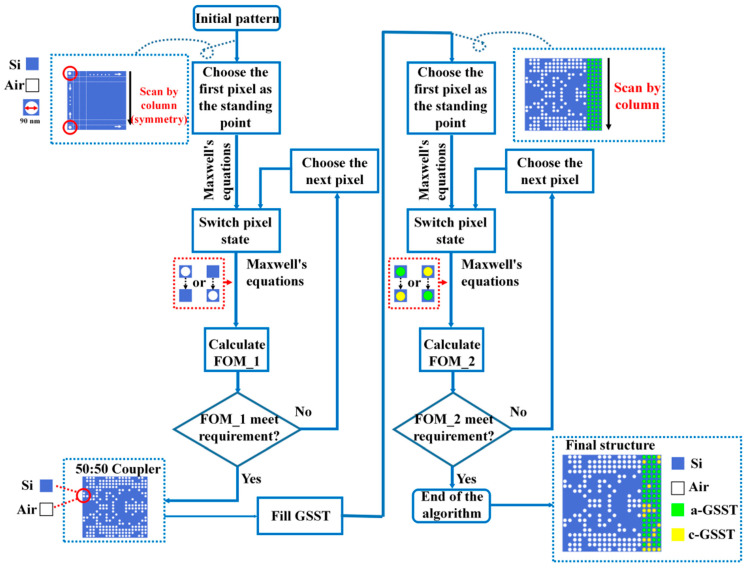
DBS algorithm optimization process. The process can be divided into two steps: first to achieve a 50:50 average power splitter and then to achieve an arbitrary power splitter with a target splitting ratio by filling GSST optimization.

The three-dimensional finite-difference time-domain (3D FDTD) method was used for simulation. The basic process of DBS algorithm optimization is shown in Figure 3. By searching the pixel state, the DBS algorithm can determine the optimal refractive index distribution in the design area to meet the functional requirements. This design approach was used in the following process. The algorithm optimization process is shown in Figure 3. In the optimized region, the pixels have binary dielectric properties: Si/air or a-GSST/c-GSST (corresponding to the logical state “0” or “1”, respectively). GSST has a small extinction coefficient, but if they are all covered in the coupling region, the transmission efficiency will be reduced to a certain extent.

**Figure 4:**
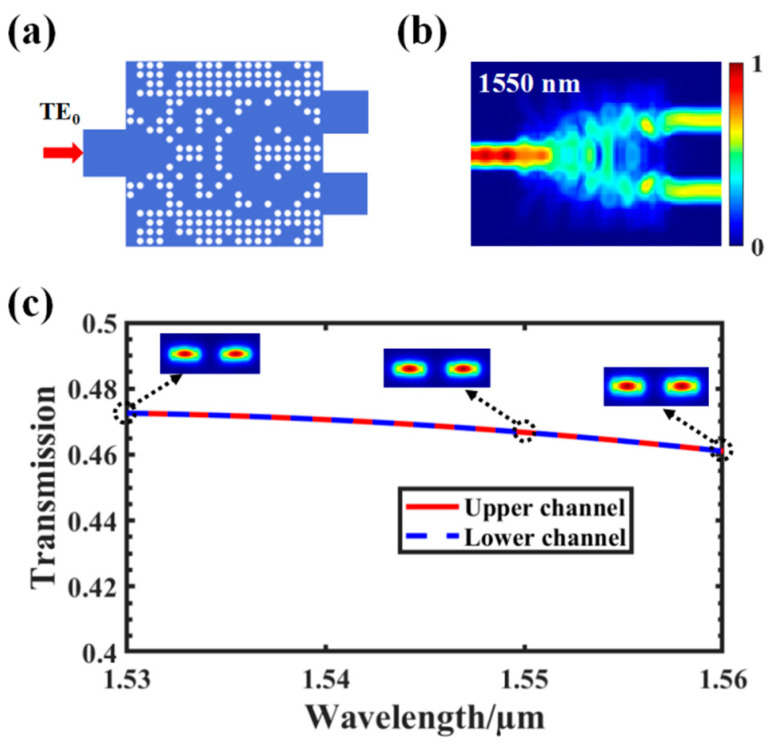
(**a**) Structure diagram of a 50:50 average power splitter; (**b**) light field distribution at 1550 nm of the upper and lower channels; (**c**) the TE_0_ mode spectral transmission curves of the upper and lower channels in the wavelength range of 1530–1560 nm were simulated, and the cross-section mode profiles of the two output channels are plotted at different wavelengths.

To design a programmable power splitter with a small footprint and high efficiency, we divided the coupling region into two parts: an air hole array and a GSST array. Basically, the optimization process contains two steps. Firstly, we set the target segmentation ratio of the two output channels as 1:1 and used the DBS algorithm to optimize the distribution of the air holes. The optimized structure could transmit power to the output channel at 50:50, and the transmission efficiency was greater than 94% as shown in Figure 4.

Then, the DBS algorithm was used to optimize the distribution of GSST, and the true value satisfying different segmentation ratios was obtained as shown in Figure 2c. This special structure could greatly reduce equipment loss and tunable complexity with an ultra-compact footprint. In this design, the true value was used to regulate the state of the GSST material in the form of an electrical pulse, as shown in Figure 2d, and the device structure conforming to the target split ratio was obtained as shown in Figure 1. The figure-of-merit (FOM) of the initial structure can be defined as:

FOM_1 = − | 0.5 − T_upper_ |(T_upper_ = T_lower_) (1)

FOM_2 = − | T_upper_ − a∙T_lower_ | (2)

where a is the split ratio of the upper and lower output channels (let the splitting ratio be a:1), and T_upper_ and T_lower_ are the average transmission efficiency of the upper and lower output channels, respectively. The FOM can be used as the quantitative evaluation function of the chip performance. The transmittance data are discrete at different wavelengths; therefore, we chose 31 data points distributed in the wavelength range of 1530–1560 nm at equal distances. Subsequently, we simulated the metamaterial structures with different refractive index distributions in an iterative process until they converged. Here, the size of the coupling region was selected as 0.48 × 2.4 μm^2^ (the selection of this size is discussed later) to achieve a precise and low-loss power split. In the ideal case, the simulation optimization value gradually approaches the target value, and the FOM should converge to a unit (FOM = 0). During the iterations, the optimization ends when the FOM does not improve further.

**Figure 5:**
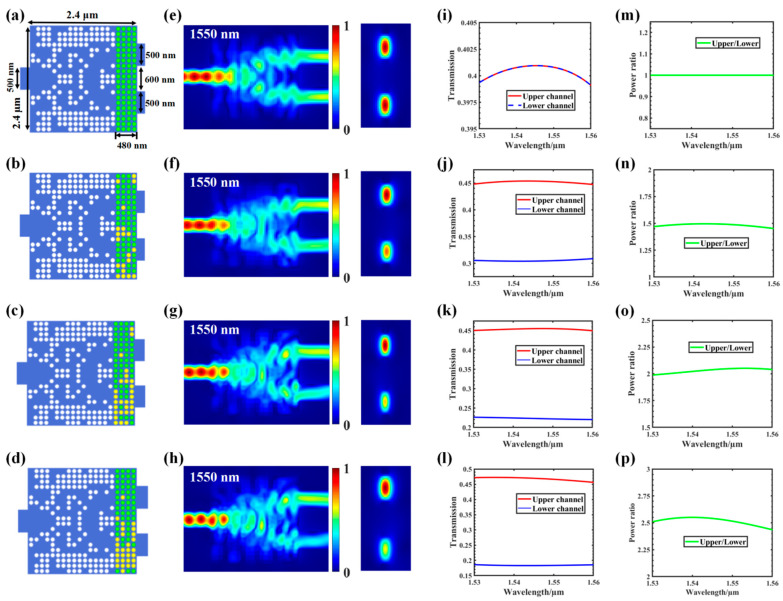
Extension to other power splitting ratios: (**a**–**d**) structure diagram of the 1 × 2 power splitter with 1:1, 1.5:1, 2:1, and 2.5:1 split ratios; (**e**–**h**) light field distribution at 1550 nm of the upper and lower channels; (**i**–**l**) transmission spectra of all tested channels; (**m**–**p**) simulated power–split ratio for the 1 × 2 splitter, where the green line indicates the simulated power ratio between the upper and lower channels in the wavelength range of 1530–1560 nm.

To verify the feasibility of our approach, we designed four power splitters with different split ratios (i.e., 1:1, 1.5:1, 2:1, and 2.5:1) for comparative analysis as shown in Figure 5. The simulation results showed that the proposed controllable power splitter usually exhibited good performance. The optical properties of GSST can be changed by applying electrical pulses without changing the structure itself. We recaptured the distribution of GSST through the optimization algorithm and, finally, obtained GSST phase distribution in line with the target split ratio.

After the optimization process was completed, we obtained a set of structures that divided the optical power in the desired proportion. Figure 5a–d shows the final structure after optimization. The light field distribution corresponding to 1550 nm and the energy intensities of the TE_0_ modulus of the cross-section of each output channel are shown in Figure 5e–h. The corresponding transmission spectra are shown in Figure 5i–l. The power–split ratios of the two output optical channels at corresponding wavelengths within the wavelength range of 1530–1560 nm was plotted as shown in Figure 5m–p.

Table 1 shows the true values corresponding to the different power–split ratios and the resulting GSST phase-distribution structure. First, we set the target split ratios (i.e., 1:1, 1.5:1, 2:1, and 2.5:1), then we obtained the true value of the input pulse through the electronic control, and finally we obtained the GSST phase distribution corresponding to the target split ratio through the impulse train. On the basis of the 3D FDTD simulations, it can be easily observed from the power–split ratio diagram that the structure calculated using the DBS algorithm had good power-splitting ability.

**Figure 6:**
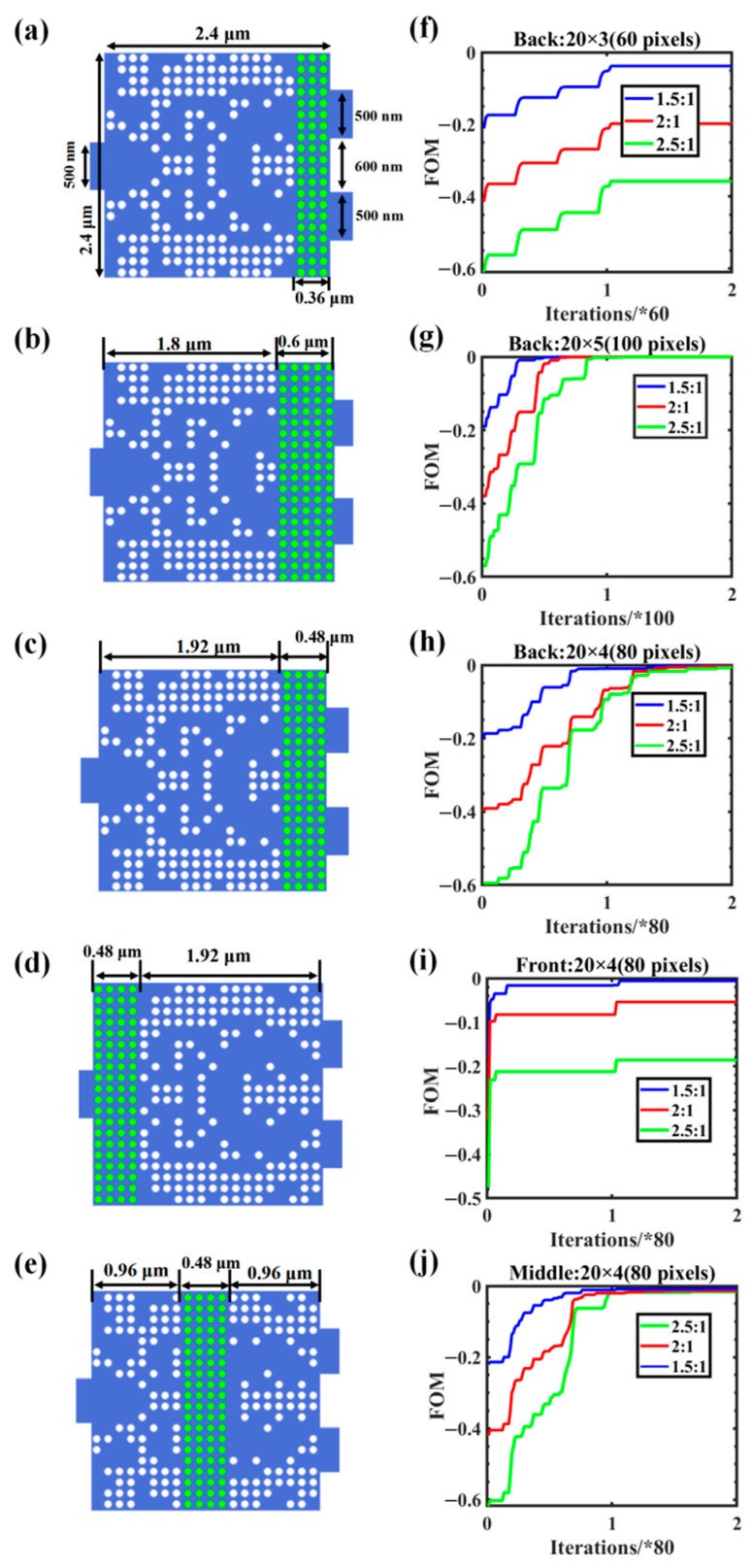
Analysis of the initial structural parameters in the second step of DBS algorithm optimization: (**a**–**e**) the initial structure of different GSST distributions; (**f**–**h**) FOM change process corresponding to pixels in the back three columns (60 pixels), the back five columns (100 pixels), and the back four columns (80 pixels) with the number of iterations; (**i**,**j**) FOM change process corresponding to pixel points in the front and middle four columns with the number of iterations (where the asterisk (*) represents the number of simulations in an iteration).

After it was confirmed that the coupling region in the proposed design could achieve any arbitrary power–split ratio, the distribution location and quantity of GSST were analyzed; this analysis is critical for minimizing the design cost and for simplifying the digital control of the ASIC. Figure 6a–c shows the limit number of GSST pixels when the target power–split ratio was satisfied. It is clear that the optimization efficiency of the pixels was low and that different split ratios can be achieved with 80 or 100 pixels. Consequently, the structures in this study were optimized with 80 pixels. In addition, we analyzed the variation in FOM with the number of iterations when the GSST was at the front, middle, and back of the coupling region as shown in Figure 6c–e. The optimized structure with GSST at the end of the coupling region had the highest efficiency. As shown in Figure 6f–j, the iterative optimization processes with different splitting ratios exhibited similar trends. We can infer the variation trend of the FOM corresponding to other higher-order splitting ratios with the number of iterations from several known splitting–ratio iteration curves shown in Figure 6. Along this optimization process, any split ratio could also be received.

## 3. 1 × 3 and 1 × 4 Arbitrary Power Splitters

**Figure 7:**
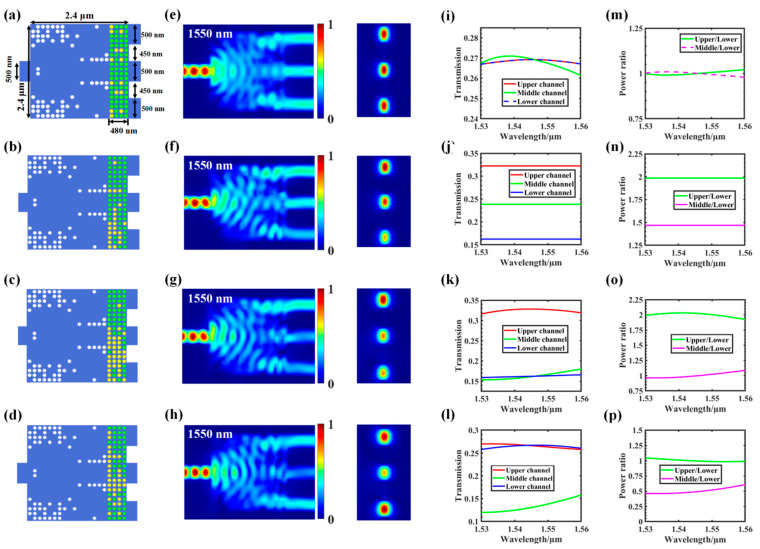
Design of a three-channel adjustable programmable power splitter: (**a**–**d**) structure diagram of a 1 × 3 power splitter with 1:1:1, 2:1.5:1, 2:1:1, and 2:1:2 split ratios; (**e**–**h**) light field distribution at 1550 nm of the upper, middle, and lower channels; (**i**–**l**) transmission spectra of all tested channels; (**m**–**p**) simulated power–split ratio for the 1 × 3 splitter, where the green line indicates the simulated power ratio between the upper and lower channels in the wavelength range of 1530–1560 nm. The purple line indicates the ratio between the middle and lower channels.

By changing the number of output channels, we can design a power splitter with additional splitting ratios. On this basis, we increased the number of output channels to three, designed a device with three split ratios, and optimized it using 3D FDTD. As shown in Figure 7a, the 1 × 3 arbitrary power splitter consisted of a 500 nm wide input waveguide; three 500 nm wide output waveguides; one coupling region of 2.4 × 2.4 µm^2^. The spacing between the output waveguides was set to 450 nm.

Figure 7 shows the performance analysis of a three-channel adjustable arbitrary power splitter by 3D FDTD. The FOM of the three-channel power splitter is:

FOM_1 = − | 0.33 − T_upper_ | −| T_upper_− T_middle_ | (T_upper_ = T_lower_) (3)

FOM_2 = − | b∙T_upper_ − a∙T_middle_ | − | c∙T_upper_ − a∙T_lower_ | − | c∙T_middle_ − b∙T_lower_ | (4)

where a–c is the split ratio of the upper, middle, and lower output channels (let the splitting ratio be a:b:c); T_upper_, T_middle_, and T_lower_ are the average transmission efficiency of the upper, middle, and lower output channels, respectively. An arbitrary power splitter with four splitting ratios is shown in Figure 7a–d. The corresponding transmission spectra are shown in Figure 7i–l. From the transmission curve, the power-splitting effect met the design objective. The light field distribution corresponding to 1550 nm and the energy intensities of the TE_0_ modulus of the cross-section of each output channel are shown in Figure 7e–h. Figure 7m–p show the power–split ratio between different channels in the wavelength range of 1530–1560 nm.

Table 2 shows the true values corresponding to different power–splitting ratios and the resulting GSST phase-distribution structure. First, we set the target splitting ratios (i.e., 1:1:1, 2:1.5:1, 2:1:1, and 2:1:2), then obtained the true value of the input pulse through electronic control, and finally obtained the GSST phase distribution corresponding to the target split ratio through the impulse train.

**Figure 8:**
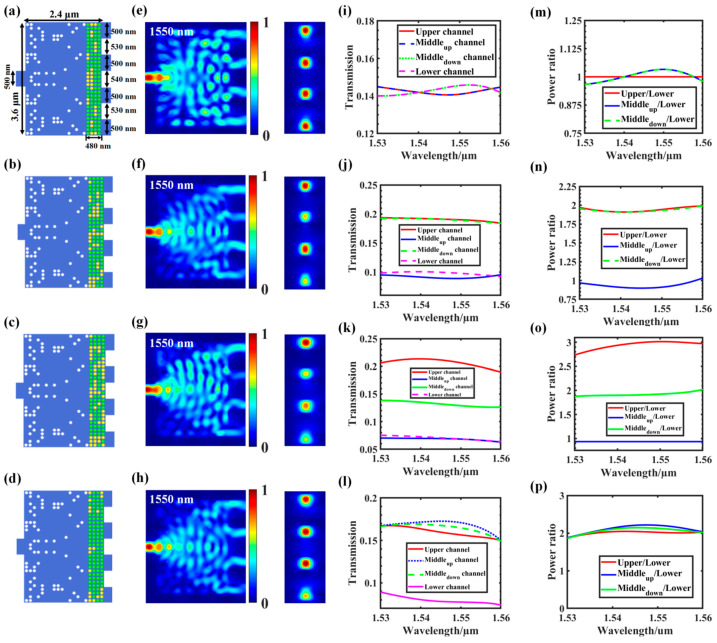
Design of a four-channel programmable arbitrary power splitter: (**a**–**d**) structure diagram of a 1 × 4 power splitter with 1:1:1:1, 2:1:2:1, 3:1:2:1, and 2:2:2:1 split ratios; (**e**–**h**) light field distribution at 1550 nm of the upper, middle_up_, middle_down_, and lower channels; (**i**–**l**) transmission spectra of all tested channels; (**m**–**p**) simulated power–split ratio for the 1 × 4 splitter, where the red line indicates the simulated power ratio between the upper and lower channels in the wavelength range of 1530–1560 nm. The blue line indicates the ratio between the middle_up_ and lower channels. The green line indicates the ratio between the middle_down_ and lower channels.

Similarly, we continued to increase the number of output channels to four to achieve an arbitrary power splitter with four split ratios. As shown in Figure 8a, the 1 × 4 arbitrary power splitter consisted of a 500 nm wide input waveguide, four 500 nm wide output waveguides, and a 2.4 × 3.6 µm^2^ coupling region with spacing between the output waveguides set at 530, 540, and 530 nm, respectively.

The FOM of the four-channel power splitter is:

FOM_1 = − | 0.25 − T_upper_ | − | 0.25 − T_middle_up_ | − | T_upper_− T_middle_up_ | (T_upper_ = T_lower_, T_middle_up_ = T_middle_down_) (5)

FOM_2 = − | b∙T_upper_ − a∙T_middle_up_ | − | c∙T_upper_ − a∙T_middle_down_ | − | d∙T_upper_ − a∙T_lower_ | − | c∙T_middle_up_ − b∙T_middle_down_ | − | d∙T_middle_up_ − b∙T_lower_ | − | d∙T_middle_down_ − c∙T_lower_ | (6)

where a–d is the split ratio of the upper, middle_up_, middle_down_, and lower output channels (let the splitting ratio be a:b:c:d). Figure 8a–d show the structure diagram of the power splitters with different splitting ratios (i.e., 1:1:1:1, 2:1:2:1, 3:1:2:1, and 2:2:2:1). The light field distribution corresponding to 1550 nm and the energy intensity of the TE_0_ modulus of the cross-section of each output channel are shown in Figure 8e–h. The corresponding transmission spectra are shown in Figure 8i–l. Figure 8m–p show the power–split ratio between different channels in the wavelength range of 1530–1560 nm.

Table 3 shows the true values corresponding to different power–split ratios and the resulting GSST phase-distribution structure. First, we set the target splitting ratios (i.e., 1:1:1:1, 2:1:2:1, 3:1:2:1, and 2:2:2:1), then obtained the true value of the input pulse through electronic control, and finally obtained the GSST phase distribution corresponding to the target splitting ratio through the impulse train. As expected, changing the number of output channels can achieve more split ratios, and precise ratios can be obtained through programming optimization. Theoretically, by regulating the phase distribution or the number of channels of the O-PCM, it can be extended to a higher split ratio and more split ratio configurations.

To demonstrate the versatility of the device, we compared the programmable device with a few reported representative arbitrary power splitters [9,20,52]. As shown in Table 4, the proposed devices had an ultra-small footprint and exhibited multi-channel and reconfigurability functions. For instance, most of the previously related works were not non-volatile and non-programmable [9,20,52]. GSST material switching between a crystalline and amorphous state gives the unique advantages of non-volatility and tunability. Here, our scheme offers non-volatility and digital programmability. Compared to the fixed power–split ratio in other studies, our device can realize an almost arbitrary split ratio through a fully digital program operation. Moreover, the footprint of our device is the smallest one in Table 4. Compared with the result in Reference [9], the footprint of our device was reduced by approximately two orders of magnitude.

## 4. Tolerance to Fabrication Errors

Over-etching and under-etching are typical errors in device manufacturing. Round holes are easier to manufacture than square holes that have too many sharp corners, but the size of the holes is not easy to control. To investigate the manufacturing tolerances of these nanostructured splitters, we simulated the effects of varying the top hole radius and silicon thickness on device performance using a 1 × 2 power splitter as an example. Here, we defined the normalized FOM error as:

FOM error = | 1 − (T_upper_/T_lower_)/a| (a = 1, 1.5, 2, 2.5) (7)

**Figure 9:**
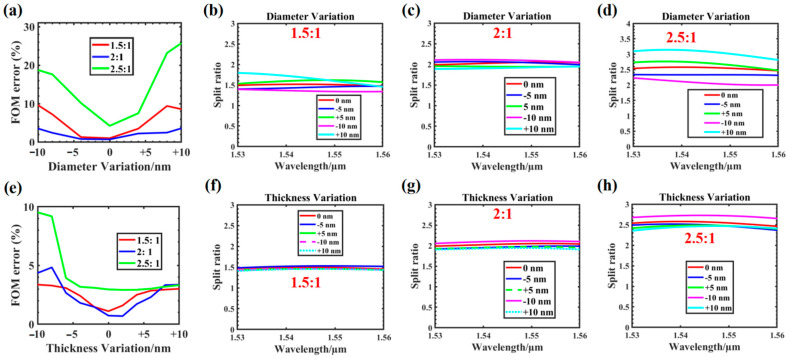
Analysis of the fabrication tolerances for the 1 × 2 photon programmable power splitter: (**a**) simulated FOM error as the hole radius varied from −10 to +10 nm; (**b**–**d**) simulated power–split ratio as the hole radius varied from −10 to +10 nm; (**e**) simulated FOM_error of the top layer of silicon as its thickness varied from −10 to 10 nm; (**f**–**h**) simulated power–split ratio of the top layer of silicon as its thickness varied from −10 to 10 nm.

It can be used to approximate the deviation between the simulated value obtained by the device and the expected value. The closer the FOM error is to 0, the smaller the deviation. Figure 9a depicts the error analysis of different power ratios, as the hole radius varied from −10 to +10 nm. Figure 9b–d show the power–split ratio curves (i.e., 1.5:1, 2:1, 2.5:1) of the round hole when its diameter changed from −10 to +10 nm in the wavelength range of 1530–1560 nm. As the aperture deviation increased, the error increased, which is a reasonable and acceptable performance [52]. It can be observed that the fabrication deviation of the device has strong robustness under the hole diameter change of ±10 nm.

Additionally, Figure 9e shows the error analysis of silicon in the −10 to 10 nm thickness range. Figure 9f–h shows the power–split ratio curves (i.e., 1.5:1, 2:1, and 2.5:1) of the round hole when the top layer silicon thickness changed from −10 to +10 nm in the wavelength range of 1530–1560 nm. In the range of −10 to 10 nm, the power–split ratio error of the output channel was very small (below 10%), indicating that the disturbance of silicon thickness had minimal influence on the transmission efficiency of the device and a good machining tolerance.

## 5. Conclusions

In summary, we proposed a reconfigurable compact photonic arbitrary power splitter based on a digital nanophotonics method and a silicon and O-PCM hybrid structure with a footprint of only 2.4 × 2.4 and 2.4 × 3.6 µm^2^. The switching between the amorphous and crystalline states of the O-PCM material GSST enabled a high-precision digital reconfiguration of the power–split ratio between the target output channels. In the bandwidth range of 1530–1560 nm, all the power ratios of 1:1, 1.5:1, 2:1, and 2.5:1 were achieved. Moreover, using the same design method, it can be extended to a multi-channel splitter to program an arbitrary splitter ratio and achieve high-precision reconfiguration. This hybrid photonics platform not only provides an effective method for designing reconfigurable ultra-small tunable photonic devices, but also offers a certain potential value for the realization of perfect optoelectronic fusion chips.

## Figures and Tables

**Table 1:** Truth tables of different split ratios in 1 × 2 power splitter, the true value ‘1’ corresponding to the yellow spots (c-GSST state).

Power Ratio	True Value	Distribution of GSST
1:1	0 0 0 0 0 0 0 0 0 0 0 0 0 0 0 0 0 0 0 0	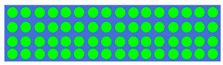
	0 0 0 0 0 0 0 0 0 0 0 0 0 0 0 0 0 0 0 0	
	0 0 0 0 0 0 0 0 0 0 0 0 0 0 0 0 0 0 0 0	
	0 0 0 0 0 0 0 0 0 0 0 0 0 0 0 0 0 0 0 0	
1.5:1	0 1 0 0 0 0 0 0 0 0 1 1 1 0 0 0 0 1 0 1	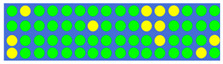
	0 0 0 0 0 0 1 0 0 0 1 1 0 0 0 0 0 1 1 1	
	1 0 0 0 0 0 0 0 0 0 0 1 0 0 0 1 1 0 0 1	
	1 0 0 0 0 0 0 0 0 0 0 0 0 0 1 0 0 0 0 1	
2:1	0 1 1 0 0 0 0 0 0 0 0 1 1 1 1 1 1 1 0 0	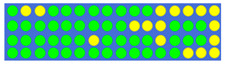
	0 0 0 0 0 0 0 0 0 1 1 1 0 0 0 1 1 1 0 1	
	0 0 0 0 0 0 1 0 0 0 0 1 0 0 0 1 0 1 0 1	
	0 0 0 0 0 0 0 0 0 0 0 0 0 1 1 1 1 1 1 1	
2.5:1	0 0 0 0 0 0 0 0 0 0 1 1 1 1 0 1 1 1 1 0	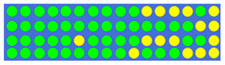
	0 0 0 0 0 0 0 0 0 0 0 0 0 0 1 1 1 1 1 0	
	0 0 0 0 0 1 0 0 0 0 1 1 1 0 0 1 1 1 1 1	
	0 0 0 0 0 0 0 0 0 1 0 0 0 1 1 1 1 1 1 0	

**Table 2:** Truth tables of 1 × 3 power splitter corresponding to different split ratios. The detailed c-GSST state distribution of four power ratio cases are presented.

Power Ratio	True Value	Distribution of GSST
1:1:1	0 0 0 0 0 0 0 0 0 0 0 0 0 0 0 0 0 0 0 0	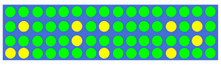
	0 1 0 0 0 1 0 1 0 0 0 0 1 0 1 0 0 0 1 0	
	0 0 0 0 0 1 0 0 0 0 0 0 0 0 1 0 0 0 0 0	
	1 1 0 0 0 0 0 1 0 0 0 0 1 0 0 0 0 0 1 1	
2:1.5:1	0 0 0 0 0 0 0 0 0 0 0 0 0 0 1 0 0 0 0 0	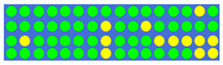
	0 0 0 0 0 0 0 1 0 0 1 0 0 0 0 0 1 1 1 0	
	0 1 0 0 0 0 0 1 0 0 0 1 1 1 1 1 1 0 0 1	
	0 0 0 0 0 0 0 1 0 0 0 0 0 0 1 1 1 1 1 1	
2:1:1	0 0 0 0 0 0 0 0 0 1 0 0 0 1 1 1 0 0 0 1	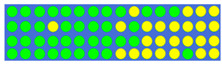
	0 0 0 1 0 0 0 0 1 0 1 1 1 1 1 1 1 1 1 0	
	0 0 0 0 0 0 0 0 0 0 1 1 1 1 1 1 1 0 0 1	
	0 0 0 0 0 0 0 0 1 1 1 1 1 0 1 1 1 1 1 1	
2:1:2	0 0 0 0 0 0 0 0 0 0 0 1 1 0 0 0 0 0 0 0	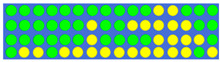
	0 0 0 0 0 0 1 0 0 1 1 1 1 1 0 0 1 0 1 0	
	0 0 0 0 0 0 1 0 0 0 0 1 1 1 1 0 1 0 0 0	
	0 1 1 0 1 1 1 1 1 1 1 1 1 1 0 1 0 0 1 0	

**Table 3:** Truth tables of 1 × 4 power splitter corresponding to different split ratios. There are four output channels and then a bigger GSST spot array (30*4) have to be used to control the split ratio.

Power Ratio	True Value	Distribution of GSST
1:1:1:1	0 0 0 0 0 0 0 0 0 0 0 0 0 0 0 0 0 0 0 0 0 0 0 0 0 0 0 0 0 0	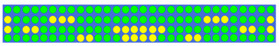
	1 0 0 0 0 1 1 1 0 0 0 0 0 0 0 0 0 0 0 0 0 0 1 1 1 0 0 0 0 1	
	1 0 1 1 0 0 0 0 0 0 0 0 1 1 1 1 1 1 0 0 0 0 0 0 0 0 1 1 0 1	
	0 0 0 0 0 0 0 0 1 1 0 0 0 1 1 1 1 0 0 0 1 1 0 0 0 0 0 0 0 0	
2:1:2:1	0 0 0 0 0 0 0 0 0 0 0 0 0 0 0 1 0 0 0 0 0 0 0 0 0 1 1 0 0 0	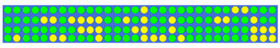
	0 0 0 0 1 1 0 1 1 1 1 0 0 1 0 1 0 0 1 0 0 0 0 1 1 0 0 1 0 0	
	0 0 0 0 0 0 0 0 1 1 1 0 0 0 1 1 0 0 1 0 0 0 0 0 0 0 0 1 1 1	
	0 1 0 0 0 1 1 0 0 0 1 0 0 0 0 1 1 1 0 1 0 0 0 0 0 0 0 1 1 1	
3:1:2:1	0 0 0 0 0 0 1 1 1 1 1 1 0 1 1 0 1 0 0 0 1 1 1 1 0 0 0 1 0 1	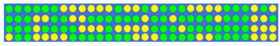
	0 0 0 1 1 0 0 1 1 0 0 0 0 0 0 1 0 0 1 0 0 0 0 1 0 0 0 1 1 0	
	0 0 0 1 0 0 0 0 1 1 1 0 1 1 1 1 0 0 0 1 0 0 0 0 0 0 0 1 1 1	
	1 0 0 1 1 1 1 0 0 0 0 0 0 0 1 1 1 0 0 0 0 1 1 0 0 0 0 1 1 1	
2:2:2:1	1 1 0 0 0 0 0 0 0 0 0 0 0 0 0 0 0 0 0 0 0 0 0 0 0 0 1 1 1 0	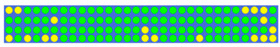
	0 0 0 0 0 1 0 0 0 0 0 0 0 0 0 0 0 0 0 0 0 0 0 0 0 0 0 0 1 0	
	0 0 0 0 0 0 0 0 0 0 0 0 0 0 0 1 0 0 0 0 0 0 0 0 0 0 0 0 0 1	
	1 0 1 0 1 1 0 0 0 0 0 0 0 0 0 1 1 0 0 0 0 1 0 0 0 0 0 1 1 1	

**Table 4:** Structural parameters and performance of arbitrary power splitters.

Refs.	[9]	[20]	[51]	This Work
Footprint	124 × 6.4 µm^2^	3.6 × 3.6 µm^2^	2.8 × 2.8 µm^2^	2.4 × 2.4 µm^2^, 2.4 × 3.6 µm^2^
Dimension of the input/output waveguide	1.6 µm	480 nm	500 nm	500 nm
Containing O-PCM	No	No	No	Yes (Ge_2_Sb_2_Se_4_Te_1_)
Operating bandwidth	1530–1570 nm	1530–1560 nm	1500–1600 nm	1530–1560 nm
Maximum number of channels	4	3	3	4
Design technology	Forward design	Inverse design	Inverse design	Inverse design
Non-volatile	No	No	No	Yes
Tunability	No	No	No	Yes
Programmable	No	No	No	Yes
Error analysis	No	No	No	Yes
Origin of results	Experiment and simulation	Experiment and simulation	Simulation	Simulation

## Data Availability

Data are available in a publicly accessible repository.
